# Draft genomes of *Diaporthe ueckeri* and *Diaporthe longicolla* strains associated with the soybean pod and grain rot disease in Brazil

**DOI:** 10.1128/mra.01225-24

**Published:** 2025-04-21

**Authors:** Hiago Antonio Oliveira da Silva, Cosma Raissa B. Nascimento, Rafael S. Anjos, Eduardo S. G. Mizubuti

**Affiliations:** 1 Departamento de Fitopatologia, Universidade Federal de Viçosa 28120, Viçosa, Minas Gerais, Brazil; University of Strathclyde, Glasgow, United Kingdom

**Keywords:** *Glycine max*, *Phomopsis*, Seed decay

## Abstract

Pod and grain rot (PGR) of soybean, also popularly known as “anomaly,” is an emergent and destructive disease of soybean in Brazil. High-quality genomes of four strains of *Diaporthe* spp., UFV-DUS-20, UFV-DUP-37, and UFV-DUG-53 of *D. ueckeri* and UFV-DLS-67 of *D. longicolla*, isolated from soybean plants showing symptoms of PGR are reported here.

## ANNOUNCEMENT

Pod and grain rot (PGR) of soybean is an emerging disease in Brazil caused by species of *Diaporthe*. The disease has caused significant reductions in yield (quantity) and quality of soybean in Brazil, the largest soybean-producing country (1).

Four genome sequences of *Diaporthe* spp. using Oxford Nanopore Technology (ONT) of the strains UFV-DUS-20, UFV-DUP-37, and UFV-DUG-53, obtained from Sorriso, Mato Grosso State, and strain UFV-DLS-67 from Rio Verde, Goiás, are reported. The strains were obtained from plant samples taken from commercial fields, and fungal isolates were obtained from symptomatic stems, pods, and grain. Fungal pycnidia or cirri were transferred from infected tissues onto potato dextrose agar medium at 25°C, and monosporic isolates were cultured in malt extract agar for 48 hours. Mycelia were harvested for DNA extraction using the Wizard Genomic DNA Purification Kit (Promega, Madison, WI, USA). The purity and quantity of DNA were assessed using a NanoDrop One/One C microvolume UV-Vis spectrophotometer (Thermo Fisher Scientific, Waltham, MA, USA) and Qubit 4 Fluorometer (Thermo Fisher Scientific). Genomic DNA was end-polished and A-tailed using the NEBnext Ultra II end repair kit (New England Biolabs, MA, USA). No fragmentation or size selection was performed. The end-prepared samples were barcoded using Blunt/TA ligase master mix (M0367L). The library was prepared by ligating sequencing adapters (SQKNBD114.24) onto double-stranded DNA fragments using NEB Quick T4 DNA Ligase (New England Biolabs, MA, USA). After purification with AMPure XP beads, the prepared library was submitted for library quality control. The library was quantified on the Qubit fluorometer and sequenced on the Nanopore PromethION 24 system (PromethION P24 and Data Acquisition Unit, ONT, Oxford, UK) using the PromethION flow cell (FLOPRO114M).

Base calling was completed with Guppy v.4.4.1 using the high-accuracy model dna_r9.4.1_450bps_hac.cfg (2). Read quality was evaluated with NanoPlot v.1.30.1 (3). Sequencing artifacts, including adapter sequences, were removed using Porechop v. 0.2.4 (4). Filtlong v.0.2.0 (5) was employed for quality and length-based filtering of reads, setting a mean quality score threshold of 8 and a minimum length of 1,000 base pairs. The processed reads were assembled using Flye v. 2.9 (6). The raw assembled contigs obtained from Flye were polished using the same ONT reads with Medaka (7) version 1.7.0 (7). The completeness of the assembly was assessed using Benchmarking Universal Single-Copy Orthologs (BUSCO) version 5.4.3 (8). Finally, gene prediction was performed using a combination of Augustus and GeneMark-ES, following the GenSAS v.6.0 eukaryotic annotation pipeline using default parameters for fungi (9).

Sequencing data revealed a genome coverage above 100× for all strains. The assembled genome sizes ranged from 58.3 to 58.5 Mb for the *D. ueckeri* strains to 62.0 Mb for the *D. longicolla* strain. The GC% content ranged from 48.52% to 52.06%, and the number of contigs ranged from 42 to 75 for the *D. ueckeri* strains, while the *D. longicolla* strain was assembled in 571 contigs. Genome completeness, assessed with BUSCO using the *Ascomycota_odb10* database, indicated at least 98% completeness of all genomes indicating proper assembly (Table 1).

Important epidemiological and plant breeding projects will benefit from the genome resources herein.

**TABLE 1:** Genome assembly statistics of the *Diaporthe* spp. strains

Strains				
Feature	UFV-DUS-20	UFV-DUP-37	UFV-DUG-53	UFV-DLS-67
Species	*D. ueckeri*	*D. ueckeri*	*D. ueckeri*	*D. longicolla*
Plant organ origin	Stem	Pod	Grain	Stem
No. reads	7,021,296	3,503,862	4,246,565	7,631,323
Read N50	1,090.0	1,167.0	1,348.0	952.0
Genome size (Mbp)	58.786	58.854	59.149	62.647
Contigs	75	42	73	571
Contig N50	2.8 Mb	8.7 Mb	4.2 Mb	436.1 Kb
GC (%)	52.17	52.18	52.06	48.52
No. CDS	17400	16363	16810	18783
BUSCO completeness (%)	99.1	99.1	99.1	98.1

## Data Availability

The Whole Genome Shotgun project has been deposited at GenBank under accession no. PRJNA1161145. The genomes are available under the accession nos. JBHILL01, JBHZEQ01, JBHZER01, and JBHILK01. SRA (raw reads) runs are available under accessions SRR31034687, SRR31037496, SRR31044245, and SRR30733617. Data annotation is available at the Zenodo repository 10.5281/zenodo.14025852.
